# SonoGuar: A Self-healing Hydrogel for Higher Fidelity Ultrasound-guided Procedure Training

**DOI:** 10.5811/westjem.48400

**Published:** 2026-05-19

**Authors:** Aswin Bikkani, Fiona Pudewa, Beshoy Gabriel, Sarah Kim, En Chang, Stephen Chai, Sreekavya Immadisetty, Xiaofeng Liu, Andrew Crouch, Steven Johnson, Jamshid Mistry

**Affiliations:** * Arrowhead Regional Medical Center, Department of Emergency Medicine, Colton, California; † California University of Science and Medicine, Department of Emergency Medicine, Colton, California; ‡ University of Southern California, Keck School of Medicine, Department of Emergency Medicine, Los Angeles, California; § University of California, Irvine, Irvine Materials Research Institute, Irvine, California; || University of Southern California, Keck School of Medicine, Department of Anesthesiology, Los Angeles, California

## Abstract

**Introduction:**

Ultrasound-compatible procedural phantoms are critical for vascular access training, but commercial models are expensive, degrade with use, and provide limited simulation of key procedural steps. Existing low-cost alternatives often lack durability and fidelity.

**Methods:**

We synthesized a novel, self-healing, ultrasound-compatible hydrogel (SonoGuar) using guar gum, borax, glycerol, oil, and water. We constructed vascular-access task trainers and evaluated SonoGuar across three domains: 1) rheological analysis of viscoelastic recovery after injury; 2) a blinded ultrasound image comparison study comparing SonoGuar against a commercial model; and 3) a prospective, randomized, single-blind crossover simulation study comparing SonoGuar to a commercial model with 41 participants (medical students, residents, and attendings).

**Results:**

At grocery store material prices, one kilogram of SonoGuar took 10 minutes of active time and about $3 to fabricate, including vessel-mimic balloons. The commercial comparison model was quoted at $350 for a single replacement insert. SonoGuar recovered its viscoelastic profile within 30 minutes of injury and demonstrated visible healing of needle tracts by five hours. In the image comparison, SonoGuar was 1.8 times more likely than the commercial model to be selected as resembling human tissue in a head-to-head comparison (64.5% SonoGuar vs 35.5% commercial model, P < .001). In simulation, 41-residents and attendings rated SonoGuar higher than the commercial phantom across all model aspects, including anatomy identification, appearance, tactile feedback, and needle visualization (average of 4.47 vs 3.77 on a 5-point Likert scale, P < .01). After cost was disclosed, all preferred SonoGuar. Medical students rated both models similarly across all model aspects and demonstrated increased levels of confidence after training with SonoGuar or commercial phantom (pre-simulation average confidence on a 5-point Likert scale of 1.46 and post-simulation average of 3.54 and 3.37, respectively, both comparisons P < .05).

**Conclusion:**

A do-it-yourself, high-fidelity hydrogel with self-healing properties, SonoGuar can be rapidly fabricated and is suitable for realistic, durable, and scalable ultrasound-guided procedure training. The low-cost hydrogel outperformed a leading commercial model in imaging realism, user confidence, and overall preference among experienced clinicians.

## INTRODUCTION

Ultrasound guidance has become the standard of care for a variety of medical procedures, allowing clinicians to visualize anatomy in real time and improve patient safety.^1^ Compared with landmark-based techniques, ultrasound-guided vascular access improves first-pass success and time to insertion, while reducing complications such as arterial puncture and pneumothorax.^2^–^7^ Simulation-based training further improves procedural competency and patient outcomes, even among experienced clinicians.^8^,^9^ As a result, professional organizations now recommend simulation-based education over traditional observational instruction, and institutions have integrated simulation into their curricula.^10^

Commercial central venous access trainers such as those offered by Blue Phantom typically use embedded tubing within proprietary, plastic-based tissue-mimicking materials (TMM). These models, or “phantoms,” reproduce the firmness of soft tissue and are shelf-stable but cost hundreds to thousands of dollars. Additionally, many commercial models degrade with use, limiting the ability to perform full procedural workflows such as cutting, dilating, and catheter insertion. Due to the high cost of replacements, trainees may be discouraged from performing these more invasive parts of a given procedure, ultimately reducing their effectiveness as training tools.^13^

To mitigate costs, a variety of low-cost or do-it-yourself (DIY) solutions have been explored. These include casting gelatin, agar, or ballistic gel as a TMM around a vessel mimic, or inserting a vessel mimic directly into tofu, pork, or chicken.^13^–^15^ Although accessible, these models are often cumbersome to prepare, short-lived, and variable in their sonographic and procedural fidelity. Some groups have pursued an intermediate approach between commercial and DIY methods, making custom polyvinyl alcohol- or polyvinyl chloride-based polymer TMMs with more favorable properties, but they require specialized equipment and more intensive synthesis.^16^,^17^

Given these limitations, there remains a need for a more suitable TMM. The ideal material would be easy to make, affordable, non-toxic, shelf-stable, and capable of tolerating repeated needle passes without generating imaging artifacts.^12^,^21^ As part of a task-trainer, the TMM should replicate the tactile feedback and sonographic appearance of human tissue while remaining compatible with a range of phantom-model architectures and 3D-printed anatomies, including those designed for vascular access.

Both human tissue and TMMs are viscoelastic, meaning they exhibit both solid and liquid behaviors when stressed, such as by a clinician’s palpation or an ultrasound probe. Rheology, the study of viscoelastic materials, has been used to characterize various tissues as well as TMMs for simulation.^18^ Two common measurements in rheology are storage modulus (G’), which quantifies the elastic, solid-like component of a material, and loss modulus (G”), which quantifies the viscous, liquid-like component. Materials with higher G’ store more energy during deformation and recover their shapes more readily, whereas materials with higher G” lose more energy as heat and deform. Designing an effective TMM requires balancing viscoelastic and imaging properties. Hydrogels such as agar and gelatin are polymeric networks that show promise because they can reproduce the mechanical and acoustic characteristics of human soft tissue.

**Population Health Research Capsule**

What do we already know about this issue?

*Ultrasound-compatible procedural phantoms are critical for vascular-access training*.

What was the research question?

*Could a homemade hydrogel with self-healing properties outperform a far more costly commercial system in imaging realism?*

What was the major finding of the study?

*A do-it-yourself vascular-access trainer that cost $3 to make outperformed a $350-dollar commercially manufactured trainer*.

How does this improve population health?

*Using do-it-yourself tissue-mimicking materials enables more effective training for invasive line and catheter placement because material cost is no longer a factor*.

In 2018, Pan et al described a self-healing hydrogel made with guar gum, glycerol, and borax, but it lacked the firmness required for use as a TMM.^19^ Building on that work, our group previously described how we modified that formulation to enhance firmness and allow for ultrasound imaging while preserving self-healing.^20^ We termed this low-cost, high-fidelity ultrasound medium “SonoGuar,” reflecting its ultrasound compatibility and guar gum base. This work expands on that initial report by validating SonoGuar for clinical simulation as a vascular access phantom through rheologic characterization, blinded ultrasound-image comparison, and a prospective, single-blind crossover simulation study enrolling medical students, residents, and attending physicians.

## METHODS

### SonoGuar Hydrogel Synthesis

The SonoGuar formulation used in our simulation study is made from five ingredients: water; canola oil; glycerol; guar gum powder; and sodium tetraborate (borax).

Synthesis Steps

Solution 1: Mix 20 grams (g) guar gum with 20 g canola oil until smooth.
Solution 2: Dissolve 20 g glycerol in 340 mL water.
Solution 3: Dissolve ~1.8 g (½ tsp) borax in 80 mL water.

**Figure 1:**
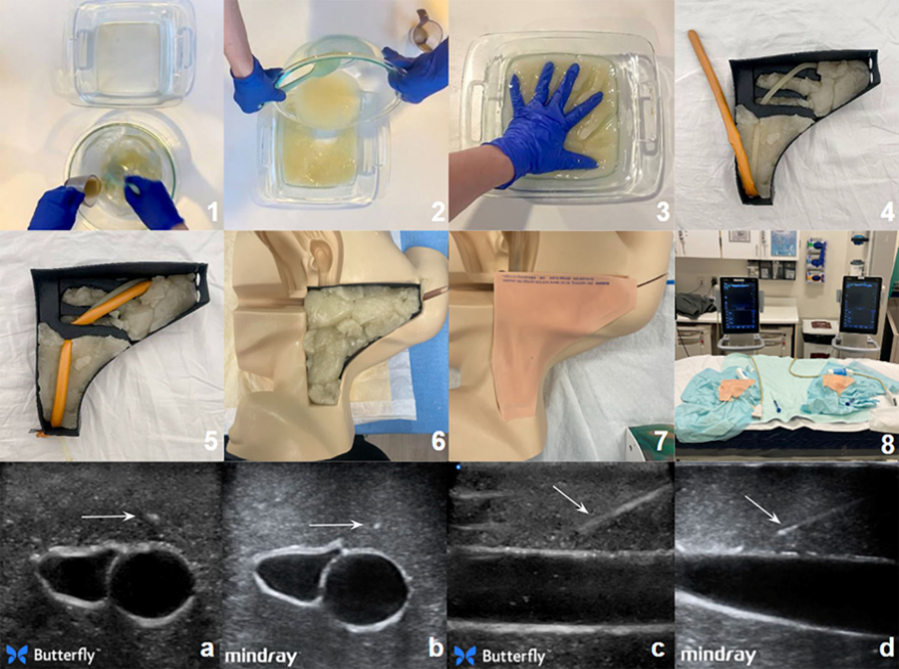
A do-it-yourself synthesis task trainer assembly, and simulation set-up. **Row 1. 1**. Stir solution 2 rapidly (pictured in glass bowl) while adding Solution 1 (pictured in measuring cup). **2**. Once opaque, immediately pour into a flat pan. **3**. Pour half of borax solution over the gel and press in with fingers; store sealed overnight. **4**. Start filling 3D mold/insert with fully cross-linked SonoGuar. Fill one small balloon with water at high pressure and one larger balloon at a lower pressure. Place small ballon over about 2 cm SonoGuar and run it under the simulated clavicle and over the ribs, securing it on the other side. **Row 2. 5**. Place large balloon directly over small balloon, running the same course, and secure the balloons on either end of the insert using a hemostat, Kelly clamp, or bobby pin. **6**. Continue packing insert with SonoGuar; leave the last 1.5 cm (in depth) of material in the container to use as a smooth slab atop the simulated internal jugular vein at the proximal portion of the insert. **7**. Place insert in task trainer. For blinding purposes, we covered it with silicone skin identical to that used on the commercial model; however, this step is not required for general use. **8**. Drape such that no part of the insert is visible; set-up is ready for resident and attending participation. **Row 3**. Demonstration of image similarity between Butterfly iQ+ (used by medical students) and Mindray Te7 (used by physicians): **a)** Butterfly iQ and **b)** Mindray Te7 axial views with needle point delineated; **c)** Butterfly iQ; and **d)** Mindray Te7 long-axis views with needle shaft delineated.

While vigorously stirring Solution 2 in a vortex, quickly pour in Solution 1 within five seconds (Figure 1). This enables the water-glycerol mix to displace the oil and hydrate the guar gum, forming a gel within ~20 seconds. The oil delays hydration, preventing clumping. Mixing must be brisk but not excessive—overmixing traps air that in turn affects imaging. This mixing step critically influences the gel’s final ultrasound characteristics, with the right amount of residual heterogeneity and air that is ideal for tissue simulation.

Immediately after mixing, pour the opaque liquid into a flat dish to maximize surface area. Do not wait for the mixture to thicken before pouring. Remove large clumps if needed. Apply half of Solution 3 to the surface and press gently; cross-linking begins instantly, changing the texture from sticky to smooth. Sticky spots indicate incomplete cross-linking and can be corrected with additional drops of Solution 3. Avoid stirring at this stage to prevent air artifact. After a few minutes, flip and apply the rest of Solution 3, again gently pressing in. Let the gel rest to finish hydration and cross-linking; about 50% of the borax is absorbed in 10 minutes, 100% within 12 hours. Gels are typically refrigerated overnight before use.

### SonoGuar Task Trainer Design

We created a 3D-printed mold to match the dimensions and anatomic landmarks (eg, clavicle) of the insert from a commercial, central venous catheter simulator (Kyoto Kagaku, Co, Ltd., Kyoto, Japan).^23^ Oblong Qualatex latex balloons sizes 350Q and 260T (Pioneer Balloon Company, Wichita, KS)) filled with water at different pressures simulated the internal jugular vein (IJV) and common carotid artery. SonoGuar was placed in the mold and around the balloon vessels; we verified vessel positioning, depth, and relative compressibility before use (Figure 1).

### Study Design

Our study included the following three components: 1) rheological analysis of the experimental material; 2) a blinded, ultrasound image-comparison study; and 3) a prospective, randomized, single-blind crossover simulation study. We enrolled 41 participants: 19 medical students (MS) without prior vascular access experience (13 MS-1, 4 MS-2, 1 MS-3, and 1 MS-4); 13 emergency medicine residents (3 postgraduate year (PGY)-1, 5 PGY-2, two PGY-3, and 3 PGY-4); three PGY-2 internal medicine residents; one PGY-1 anesthesia resident; and five attending physicians in emergency medicine. The surveys used a 5-point scale to assess confidence (six items) and model evaluation (four items).

### Rheological and Optical Characterization

We measured viscoelastic properties using a Discovery HR-2 Hybrid Rheometer (TA Instruments, New Castle, DE) at the Irvine Materials Research Institution (IMRI). Storage (G’) and loss (G”) moduli were recorded under oscillatory shear at 1 hertz with an axial force of 0.5 Newtons. Self-healing was assessed by recombining cut samples, with measurements taken at 5, 15, and 30 minutes. We performed optical microscopy at IMRI using an AMScope SM-3T microscope (United Scope, LLC, Irvine, CA) on a simulation sample of SonoGuar and a sample that we dyed blue using Favorite Day food coloring (Target Corporation, Minneapolis, MN).

### Image Comparison Study

In the blinded, pairwise, ultrasound image comparison, participants viewed 15 randomized ultrasound image pairs of either SonoGuar, a commercial model, or human tissue. For each pair, participants selected the image that best resembled human tissue. These included transverse axis view of the IJV and common carotid artery with needle point 1) abutting the IJV; 2) within the IJV lumen; or 3) not visible, as well as a longitudinal axis view of the IJV with the needle; 4) abutting the IJV; or 5) within the lumen. We standardized commercial trainer and SonoGuar images by ultrasound settings, acquired with the Butterfly iQ+ (Butterfly Network, Inc, Burlington, MA). All 41 participants completed this prior to hands-on simulation.

### Crossover Simulation Study

In the crossover simulation study, participants compared the SonoGuar task trainer to the commercial model Kyoto Kagaku CVC Insertion Simulator III. To ensure blinding, both models were covered with identical silicone skins and draped to conceal the TMM (Figure 1). No participants had prior experience with either model.

Simulation sessions were held at the California University of Science and Medicine Clinical Skills Department for medical students, where Butterfly iQ+ handheld ultrasound probes were available (vascular setting, 3.5-cm depth). Resident and attending sessions were conducted in the Arrowhead Regional Medical Center emergency department using cart-based Mindray TE7 machines (Shenzhen Mindray Bio-Medical Electronics Co., Ltd., Shenzhen, People’s Republic of China) (vascular setting, 3.5-cm depth). On both devices, SonoGuar and the commercial phantom appeared similar in imaging quality (Figure 1).

Medical students received a 30-minute instructional session covering central venous access, ultrasound-guidance principles, and procedural steps. All residents had completed at least one central line on a real patient, with most having completed > 10. Physicians outperformed students in human tissue recognition (81.8% vs 63.7%, *P* < .001). Participants then performed ultrasound-guided venous access on both models using an 18-gauge needle and a 5-cc syringe. The order of model use was randomized. Because the commercial model could not tolerate multiple full-procedure attempts (eg, guidewire insertion, dilation, catheter placement), the simulation was concluded upon successful venous access only. Participants were informed in advance that performance metrics (eg, time to insertion) were not being evaluated.

Each participant had five minutes per model to achieve venous access. Following each attempt (or time out), participants completed a survey assessing user confidence and model performance using a Likert scale before crossing over to the next model. After using both models, they rated their overall experience and preference. At the conclusion of their session, participants were invited to interact with the raw hydrogel materials and a freestanding SonoGuar phantom in a separate demonstration area.

### Statistical Analysis

As this was a pilot feasibility study, and as we were uncertain of potential effect sizes, we did not perform power calculations. Pairwise image rankings were modeled using a Bradley-Terry framework.^22^ We analyzed confidence and model assessment data using Wilcoxon signed-rank tests and SPSS Statistics v28.0.1.0. (IBM Corp., Armonk, NY). Significance was defined as two-sided *P* < .05.

## RESULTS

### Self-healing

**Figure 2:**
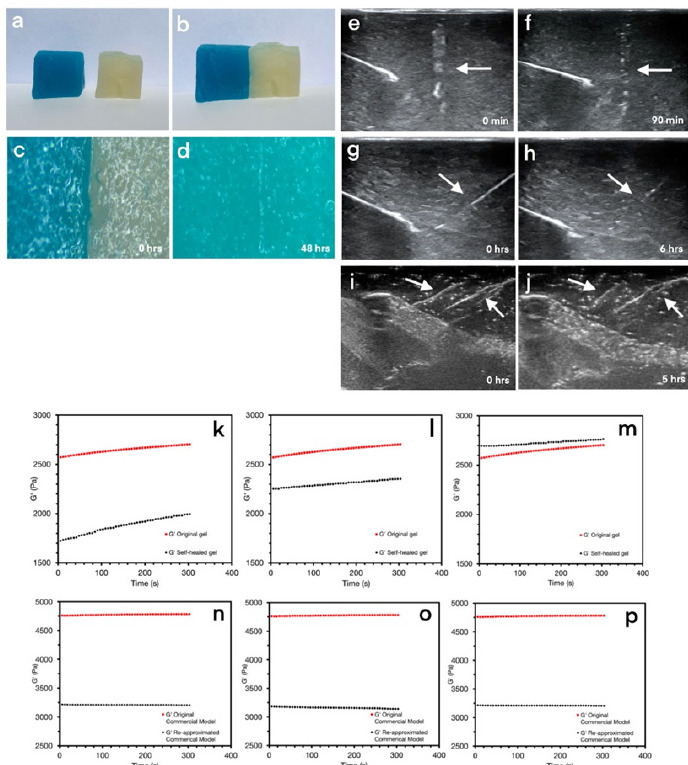
Visual and rheological evaluation of self-healing SonoGuar hydrogel: **a)** two pieces from original hydrogel with and without dying using blue food coloring; **b)** two hydrogel pieces in contact at room temperature for 48 hours; **c–d)** optical microscopy images showing changes at the healing interface over 48 hours; **e–f)** ultrasound images of SonoGuar immediately after injury and after specified time (white arrows indicate post-injury artifact). An 18-gauge needle in long-axis view is present at the left of images **e, f, g, h** as a marker: **e)** SonoGuar immediately after being cut through by #10 scalpel and **f)** 90 minutes after; **g)** SonoGuar immediately after 18-gauge needle puncture and **h)** 6 hours after; **i)** SonoGuar with a variety of echotextures, showing two artifact tracts immediately after puncture and **j)** 5 hours after. **(k–m)** Storage modulus (G’, in Pascals) of original and self-healing hydrogel over time (in seconds) at three different intervals, **k)** 5 minutes, **l)** 15 minutes, and **m)** 30 minutes; **(n–p)** storage modulus (G’, in Pascals) of original and re-approximated commercial model material over time (in seconds) at three different intervals; **j)** 5 minutes, **k)** 15 minutes, and **l)** 30 minutes. There was no change in G’ of the re-approximated material.

Rheometry showed SonoGuar’s mean storage modulus (G’) as 2,700 Pascal (Pa) and loss modulus (G”) as 470 Pa under 1 hertz oscillatory shear. Viscoelastic properties recovered fully within 30 minutes at room temperature. Optical microscopy confirmed binding and dye transfer across the healing interface. Needle-pass artifacts began resolving within five hours and progressed over time (Figure 2). The commercial model’s mean storage modulus (G’) was 4,750 Pa under 1 hertz oscillatory shear. The G’ of the re-approximated commercial phantom material remained constant (Figure 2).

### Image Comparison

Using the Bradley-Terry model on pooled student and physician data, human tissue images were selected in 67.4% of comparisons, followed by SonoGuar (22.9%) and the commercial model (9.7%). When directly compared to the commercial model, SonoGuar was chosen over the commercial model in 64.5% of cases, making it 1.8 times more likely to be identified as human-like (64.5% SonoGuar vs 35.5% commercial model, *P* < .001). Selection rates were not significantly different between students and physicians (69% vs 67%).

### Crossover Simulation

Physicians strongly preferred SonoGuar over the commercial trainer, with 90.1% favoring it (*P* < .001). After learning about cost differences (~$5 vs $350), all physicians selected SonoGuar. Wilcoxon signed-rank analysis showed higher confidence scores with SonoGuar across all procedural domains: anatomy identification; needle visualization/tracking; access; and needle placement (*P* = .01). SonoGuar also outperformed the commercial model in visual realism, tactile feedback, and ease of needle visualization (*P* = .04) (Table 1).

Medical students rated both models similarly across all domains. No preference was found in realism or usability. After cost information was provided, 84.2 % of students preferred SonoGuar (*P* < .01) as an ultrasound phantom. Significant improvements in student-reported confidence were observed after the training session across all competencies (means increasing from 1.37–1.63 to 3.21–3.68; *P* < .001), with neither phantom outperforming the other in the magnitude of confidence gain (Table 2).

## DISCUSSION

**Figure 3:**
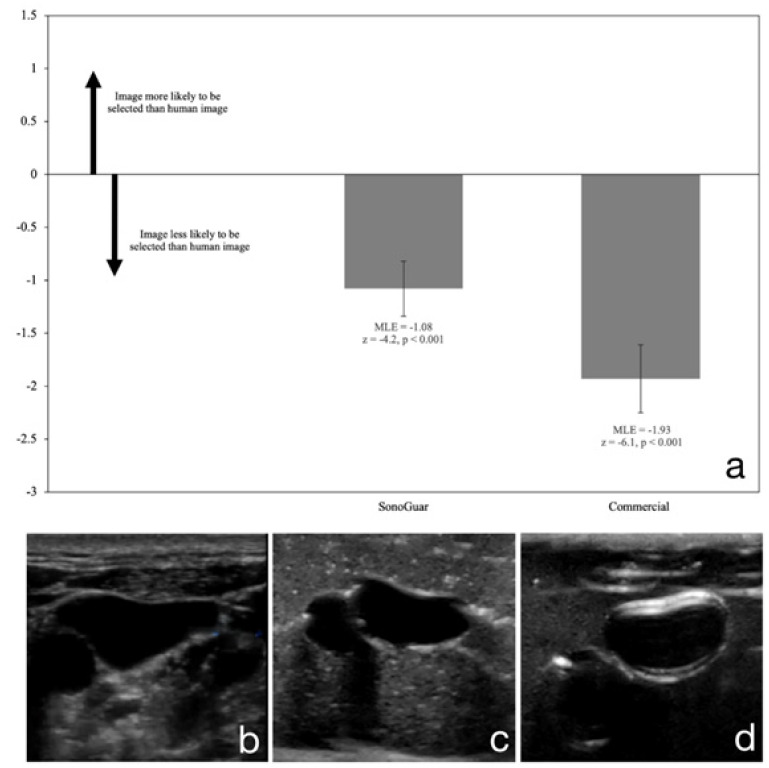
Pairwise imaging comparison of human tissue, commercial model, and homemade SonoGuar: a) Bradley-Terry model results showing the maximum likelihood estimate (MLE) of selection likelihood compared to a reference human tissue image. Both SonoGuar and the commercial model were less likely to be selected compared to the human image. SonoGuar was more likely to be selected compared to the commercial model (SonoGuar MLE = −1.08, z = −4.2, P < .001; commercial MLE = −1.93, z = −6.1, P < .001); b) ultrasound image of human tissue; c) ultrasound image of SonoGuar; and d) ultrasound image of commercial model.

Our do-it-yourself SonoGuar phantom outperformed the commercial phantom across all three study domains. Rheological testing showed full recovery of mechanical properties after disruption, unlike the commercial model, which did not return to baseline. Real-world procedural experience likely allowed residents and attendings to distinguish human tissue 28.4% more often than medical students in the imaging comparison. Subsequently, they strongly preferred the SonoGuar phantom to the commercial model across all procedural domains (Figure 3). Taken together, these findings suggest that users with more extensive procedural and ultrasound experience find SonoGuar to be a superior simulation experience.

Medical students rated SonoGuar comparably to the commercial model for simulation (Table 1), found it more life-like in the imaging comparison (Figure 3), and preferred it for cost. Even when considered as a training model for medical students, who in our study had no strong preference for either model, SonoGuar demonstrated comparable performance to commercial models (Table 2). Moreover, the advantages observed by residents and attendings would apply to trainees of all skill levels, independent of their ability to appreciate differences between models compared to real-life practice. Our study focused on SonoGuar’s procedural fidelity, but its other advantages warrant discussion.

### Synthesis, Storage, Ease-of-use, and Cost

Do-it-yourself task trainer materials like gelatin and agar require heating, molding, and cooling. Other materials explored in the literature, like polyvinyl alcohol- or polyvinyl chloride-based polymer, require high temperatures up to 170 °C and less accessible equipment, such as fume hoods.^18^ Gelatin and agar have limited refrigerator life and can fracture easily.^14^,^24^ Meat and tofu models spoil more rapidly and carry ethical and hygiene concerns. These options are all generally single use.^13^,^21^ Ballistics gel requires an initial casting but is relatively more robust.^25^

SonoGuar is mixed at room temperature using inexpensive, accessible ingredients and no specialized equipment. In our experience throughout the study period, it lasted for months at refrigerator temperature without apparent microbial degradation or changes to appearance, texture or odor. SonoGuar does not require any special handling. The amount of hydrogel used for one task trainer in our study costs approximately $3 at local grocery store prices, or $1 if ingredients are purchased in bulk. This is 4–10 times cheaper than one source of ballistics gel.^26^ Balloons cost approximately $0.06 each. Appendix A contains a full cost breakdown with links.

### Durability and Fidelity

Gelatin and agar fracture easily, and embedded vessels are difficult to replace without damaging the surrounding tissue-mimicking material.^13^,^15^ Commercial models also have limited longevity. Their TMMs are rated for a limited number of needle passes before suffering physical and imaging degradation. Up-front and replacement costs are high. The Kyoto Kagaku CVC III model recommends the use of 23-gauge needles or smaller to limit damage. We observed the accumulation of needle pass artifacts in this model, consistent with prior reports across commercial brands.^12^

High-use areas, particularly overlying cannulation sites, deteriorate with cutting, dilation, and catheter placement. Even “self-healing” TMMs, such as the Blue Phantom ultrasound training simulator (Elevate Healthcare, Sarasota, FL), explicitly warn against the use of scalpels or cutting instruments. While “self-healing” tissues may permit a higher number of needle passes before visible artifact is introduced, they do not permit truly invasive procedural simulation. Additionally, commercial systems do not allow independent replacement of vessel mimics. Vessels are permanently embedded, requiring complete insert replacement. To improve durability, these vessels are built thick, reducing compressibility and contributing to imaging artifacts such as the double-lumen effect (Figure 3d). Some models replace tubing with hollow channels, but these offer no tactile feedback on entry.^11^

SonoGuar addresses these limitations directly. It tolerates repeated needle passes, cutting, and catheterization without lasting artifact. The gel can be rotated, reshaped, or replaced as needed and does not adhere to its container. Vessel mimics are easily swapped without damaging the surrounding gel. We used thin-walled balloon vessels, which—unlike the rigid tubing in the commercial comparator—better replicate key behaviors of human veins, including compressibility and “tenting” against the needle, an important visual cue during ultrasound-guided access.^27^,^28^ While balloon mimics may lose fluid over time, the gel’s tamponade effect minimizes this, and fluid can be easily replaced.

### Tunability, Adaptability and Scalability

Most models—DIY or commercial—are static once cast. Adjusting targets, replacing vessels, or customizing anatomy typically require complete recasting or purchasing new units. SonoGuar is moldable, self-healing, and fully customizable. It conforms to its container, binds around inserted structures, and supports modular training using 3D-printed anatomies. Ultrasound characteristics can be tuned during synthesis for novice or advanced learners. Anatomic features, including fascial planes, muscle bellies, tendons, or bony landmarks, can be included. Instructors can rapidly reset, reposition, or modify targets in real time. The same amount of gel can be used to simulate different procedures during different sessions.

A low-cost, durable, high-fidelity vascular access model also supports broader integration into institutional training. Some programs conduct large-scale onboarding simulations involving multiple simultaneous stations, where the up-front cost of multiple commercial simulators would be prohibitive. Programs have implemented institution-wide mastery learning curricula for central venous catheterization and adopted just-in-time training strategies, where task trainers are available year-round without requiring a designated facilitator.^29^,^30^

## LIMITATIONS

Our sample size of 41 participants was small, and because this was designed as a feasibility study we did not perform an a priori power calculation. Outcomes were based on self-reported survey data rather than objective performance measures. Survey items were developed with attending input and reviewed by peers for content validity, but they were not formally psychometrically validated. In addition, some overlap likely existed between needle-related parameters, particularly as participants retrospectively reflected on their simulation experience. Because of this and because we did not adjust for multiple comparisons, the overall simulation experience rating may represent the most robust comparative measure. Our commercial phantom comparator was limited to a single phantom (the Kyoto Kagaku CVC III); other widely used brands such as Blue Phantom were not included. Furthermore, different ultrasound machines were used at the two study sites (Butterfly iQ + vs Mindray Te7), which may have introduced confounding, although we found the images (Figure 1) and simulation experience comparable.

SonoGuar’s balance of storage and loss modulus renders it less stiff and more moldable than the commercial TMM. In some cases, this led to the probe “sinking” with excessive pressure. This did not appear to influence whether participants visualized the needle or gained access during the simulation. A stiffer gel can be made by decreasing the water content. Finally, as SonoGuar’s healing allows for compatibility with different vessel mimics, we used latex balloons, which may have contributed to perceived fidelity relative to the commercial vessel mimics.

## CONCLUSION

Do-it-yourself SonoGuar is a readily accessible, self-healing, hydrogel that outperforms a leading commercial central venous access simulator in imaging quality and procedural realism—at less than 1% the cost. It allows for full procedural simulation and offers strong advantages in terms of ease of synthesis, adaptability, and durability. Future work will explore freeze-thawing and use of ingredients such as isopropyl alcohol for long-term shelf stability and microbial resistance, as well as explore modifications to tailor SonoGuar’s stiffness and appearance for different procedural applications.

## Supplementary Information



## Figures and Tables

**Table 1:** Participant ratings of do-it-yourself SonoGuar vs a commercial ultrasound model across various procedural aspects.

Model Aspect	Residents and Attendings			Medical students		
	
	SonoGuar (Median [IQR])	Commercial (Median [IQR])	*P*-value	SonoGuar (Median [IQR])	Commercial (Median [IQR])	*P*-value
Vascular anatomy identification	5.0 (4.75–5.0)	5.0 (3.0–5.0)	< .01^*^	4.0 (3.0–5.0)	4.0 (3.0–5.0)	.97
Needle visualization	5.0 (4.0–5.0)	5.0 (3.0–5.0)	< .01^*^	4.0 (3.0–5.0)	4.0 (3.0–4.0)	.96
Needle tracking	5.0 (4.0–5.0)	4.5 (3.0–5.0)	.01^*^	3.0 (3.0–4.0)	4.0 (3.0–4.0)	.92
Vessel access	5.0 (4.75–5.0)	4.5 (3.0–5.0)	< .01^*^	4.0 (3.0–4.0)	3.0 (3.0–4.0)	.60
Needle placement confirmation	5.0 (4.75–5.0)	4.5 (3.0–5.0)	.01^*^	4.0 (3.0–4.0)	4.0 (2.0–4.0)	.25
Needle placement improvement	5.0 (4.75–5.0)	4.5 (3.0–5.0)	< .01^*^	4.0 (3.0–4.0)	3.0 (3.0–4.0)	.59
Visual appearance compared to human tissue	4.0 (3.75–5.0)	3.0 (2.0–4.0)	< .01^*^	4.0 (3.0–4.0)	4.0 (3.0–4.0)	.76
Tactile feedback compared to human tissue	4.0 (3.0–5.0)	3.0 (2.75–4.0)	< .01^*^	4.0 (3.0–4.0)	4.0 (3.0–4.0)	.83
Ease of needle visualization compared to human tissue	4.0 (3.0–5.0)	4.0 (2.0–4.25)	.04^*^	4.0 (3.0–4.0)	4.0 (3.0–4.0)	.67
Overall model experience	4.0 (4.0–5.0)	3.0 (2.75–5.0)	< .01^*^	4.0 (3.0–5.0)	4.0 (4.0–5.0)	1.00

Data are presented as mean (SD). Scores from residents, attendings, and medical students are shown separately.

Asterisks (^*^) indicate statistically significant differences (P < .05) based on the Wilcoxon signed-rank test.

*IQR*, interquartile range.

**Table 2:** Pre- and post-simulation confidence ratings of medical students after using the do-it-yourself SonoGuar and a commercial ultrasound model to practice vascular access skills.

Model Aspect	Pre-Simulation (Median ([QR])	Post-Simulation (Median [IQR])			

		SonoGuar	*P-*value	Commercial	*P*-value
Confidence in vascular anatomy identification	1 (1–2)	4 (3–4)	< .001^*^	4 (3–5)	< .001^*^
Confidence in needle tracking	1 (1–2)	3 (3–4)	< .001^*^	4 (3–4)	< .001^*^
Confidence in gaining access to vessel	1 (1–2)	4 (3–4)	< .001^*^	3 (3–4)	< .001^*^
Confidence in confirming needle placement	1 (1–2)	4 (3–4)	< .001^*^	4 (2–4)	< .001^*^
Confidence in improving needle placement	1 (1–2)	4 (3–4)	< .001^*^	3 (3–4)	< .001^*^

Scores reflect perceived confidence in performing key vascular access skills and are presented as mean (SD). Asterisks (^*^) denote significant increases in confidence from baseline (P < .05) based on the Wilcoxon signed-rank test.

*IQR*, interquartile range.
