# Supplementary material for: SonoGuar: A Self-healing Hydrogel for Higher Fidelity Ultrasound-guided Procedure Training
Source: West J Emerg Med. 2026 May 19;27(3):735–44. doi: 10.5811/westjem.48400 (PMC13246207; doi:10.5811/westjem.48400)
Supplement: Supplementary file 1 [file wjem-27-735-s001.docx]

Appendix A. Estimated Cost of SonoGuar Materials

**Recipe scale:** One batch consists of 20 g guar gum powder, 20 g canola oil, 20 g glycerol, 340 mL water, and borax solution made from approximately 1.8 g sodium tetraborate in 80 mL water. Water was assigned no material cost.

| **Ingredient** | **Amount** | **Small retail source** | **Small retail cost** | **Amazon / bulk source** | **Amazon / bulk cost** |
| --- | --- | --- | --- | --- | --- |
| Guar gum powder | 20 g / batch | Sprouts: Bob's Red Mill Guar Gum, 8 oz, $8.49 [Source link](https://shop.sprouts.com/store/sprouts/products/18025344-bob-s-red-mill-guar-gum-8-0-oz) | ~$0.75 / batch | Amazon: Generic Guar Gum Powder, 50 lb, $119.95 [Source link](https://www.amazon.com/Generic-Guar-Gum-Powder-Thickener/dp/B0D96T6NT8) | ~$0.11 / batch |
| Canola oil | 20 g / batch (~0.74 fl oz) | Walmart: Great Value Canola Oil, 48 fl oz, $3.76 [Source link](https://www.walmart.com/ip/10450988) | ~$0.06 / batch | Amazon Grocery: Canola Oil, 48 fl oz [Source link](https://www.amazon.com/Amazon-Brand-Happy-Canola-Ounces/dp/B07P5V4DCR) | ~$0.06 / batch |
| Glycerol | 20 g / batch | Walmart: Equate Vegetable Glycerin, 6 fl oz, $5.76 [Source link](https://www.walmart.com/ip/Equate-No-Scent-Liquid-Glycerin-Oil-USP-6-fl-oz/612598672?classType=VARIANT&athbdg=L1200&from=/search) | ~$0.52 / batch | Amazon: US+ Vegetable Glycerin, 1 gallon, $29.99 [Source link](https://www.amazon.com/100-Pure-Vegetable-Glycerin-Pharmaceutical/dp/B0BR99MF15) | ~$0.12 / batch |
| Borax powder | ~1.8 g / batch | Walmart: 20 Mule Team Borax, 76 oz, $5.97 [Source link](https://www.walmart.com/ip/20-Mule-Team-Borax-All-Natural-Laundry-Booster-76-oz/20850525?classType=VARIANT&athbdg=L1102) | ~$0.005 / batch | Same 76 oz box; already effectively bulk | ~$0.005 / batch |
| Water | 420 mL / batch |  | $0.00 |  | $0.00 |
| **Total hydrogel only** |  |  | **~$1.34 / batch** |  | **~$0.30 / batch** |

**Vessel-mimic balloons**

| **Vessel mimic** | **Amount per trainer** | **Amazon source** | **Cost** |
| --- | --- | --- | --- |
| 260 balloon | 1 balloon / trainer | Amazon: 100Pcs 260 Balloons, $5.29 [Source link](https://www.amazon.com/Balloons-Thickening-Twisting-Christmas-Decorations/dp/B09DYKFSQ8) | ~$0.05 / balloon |
| 360 balloon | 1 balloon / trainer | Amazon: 200Pcs 360 Balloons, $14.34 [Source link](https://www.amazon.com/Balloons-Twisting-Assorted-Christmas-Decoration/dp/B0D3HQHL5M) | ~$0.07 / balloon |

**Cost interpretation:** Each task-trainer insert used approximately two recipe-scale batches plus one 260 balloon and one 360 balloon. The per-insert totals were rounded up to approximately $3 using small-retail pricing and approximately $1 using Amazon/bulk pricing to account for shipping, tax, price variation, and transfer loss.

**Screenshots**

| 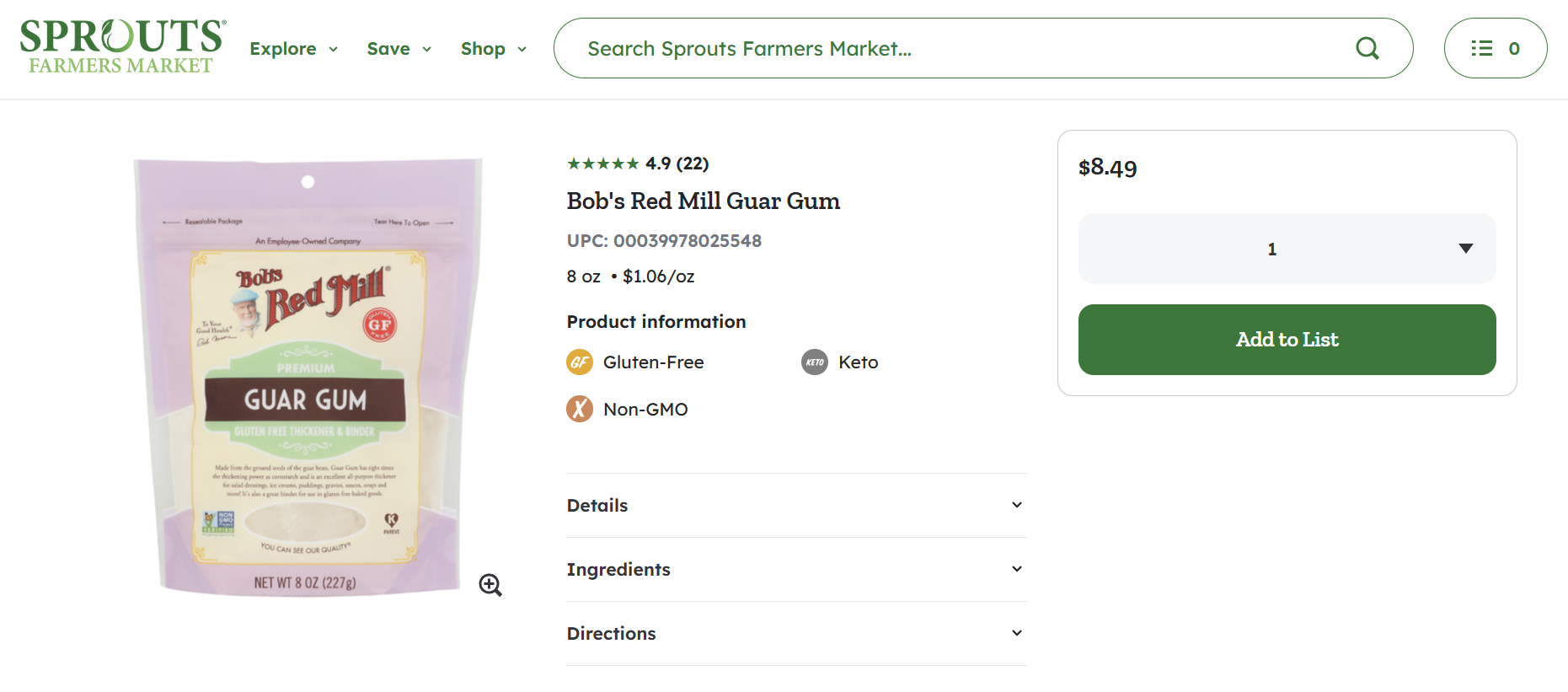 | 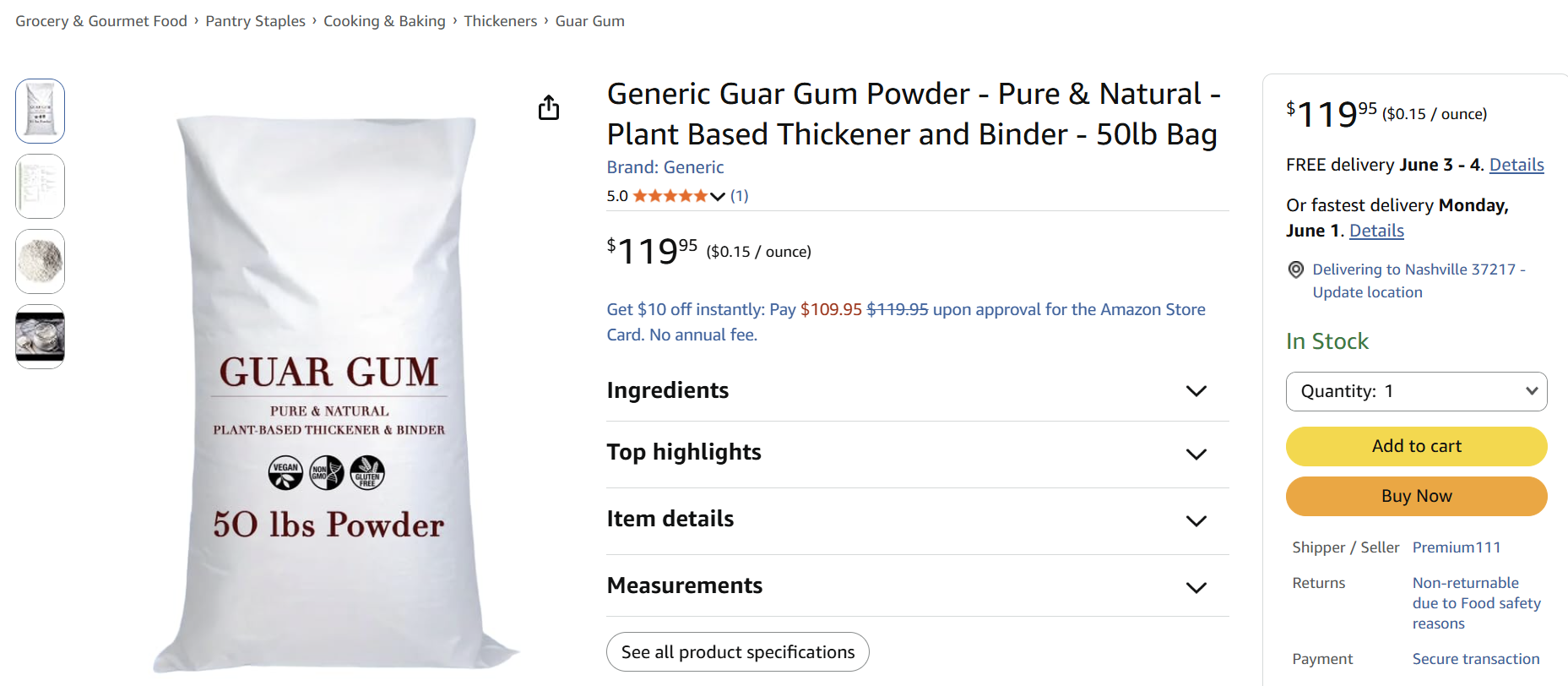 |
| --- | --- |
| **A. Guar gum, small retail: Sprouts Bob's Red Mill Guar Gum, 8 oz, $8.49** | **B. Guar gum, Amazon/bulk: Generic Guar Gum Powder, 50 lb, $119.95** |

| 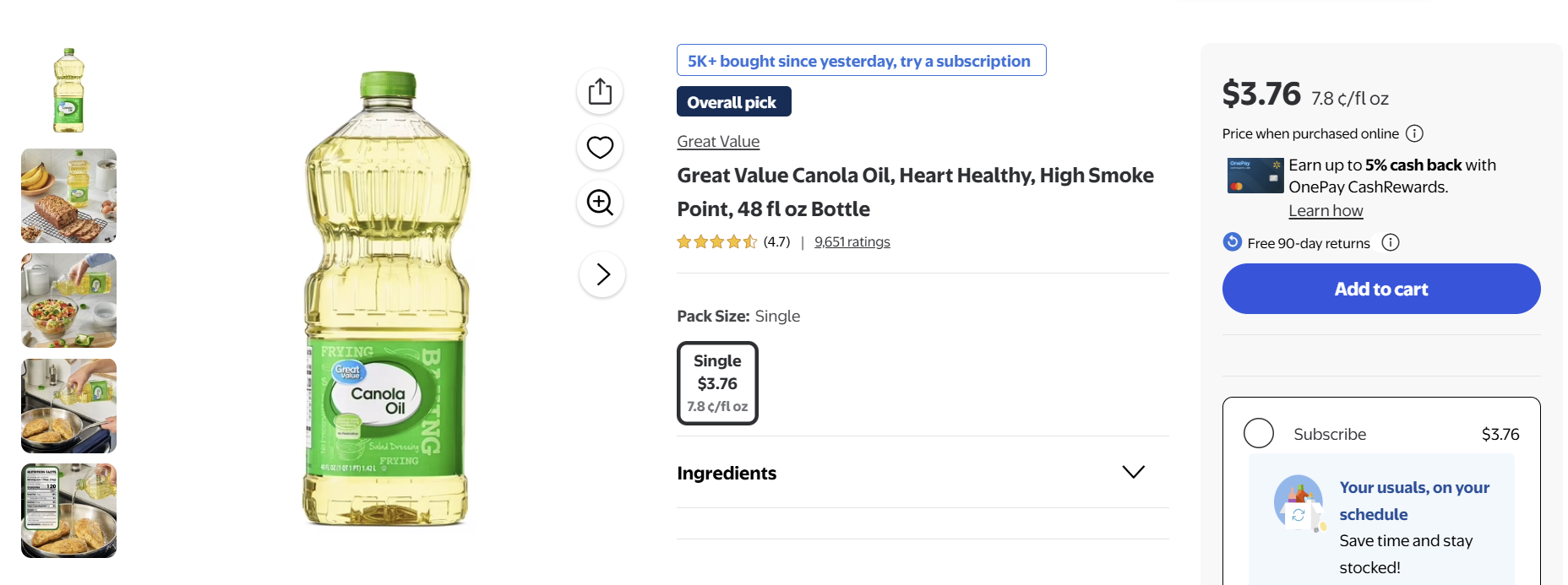 | 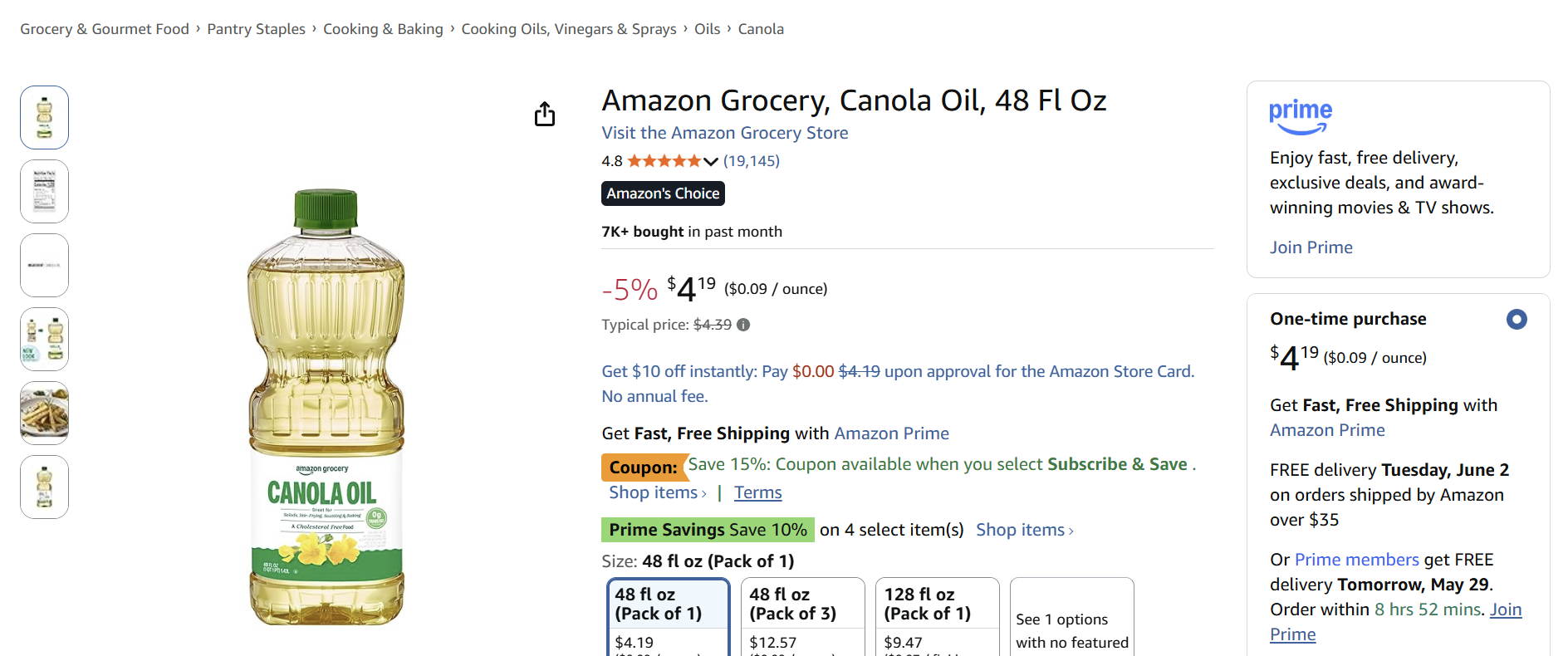 |
| --- | --- |
| **C. Canola oil, small retail: Walmart Great Value Canola Oil, 48 fl oz, $3.76** | **D. Canola oil, Amazon/bulk: Amazon Grocery Canola Oil, 48 fl oz, $4.19** |

| 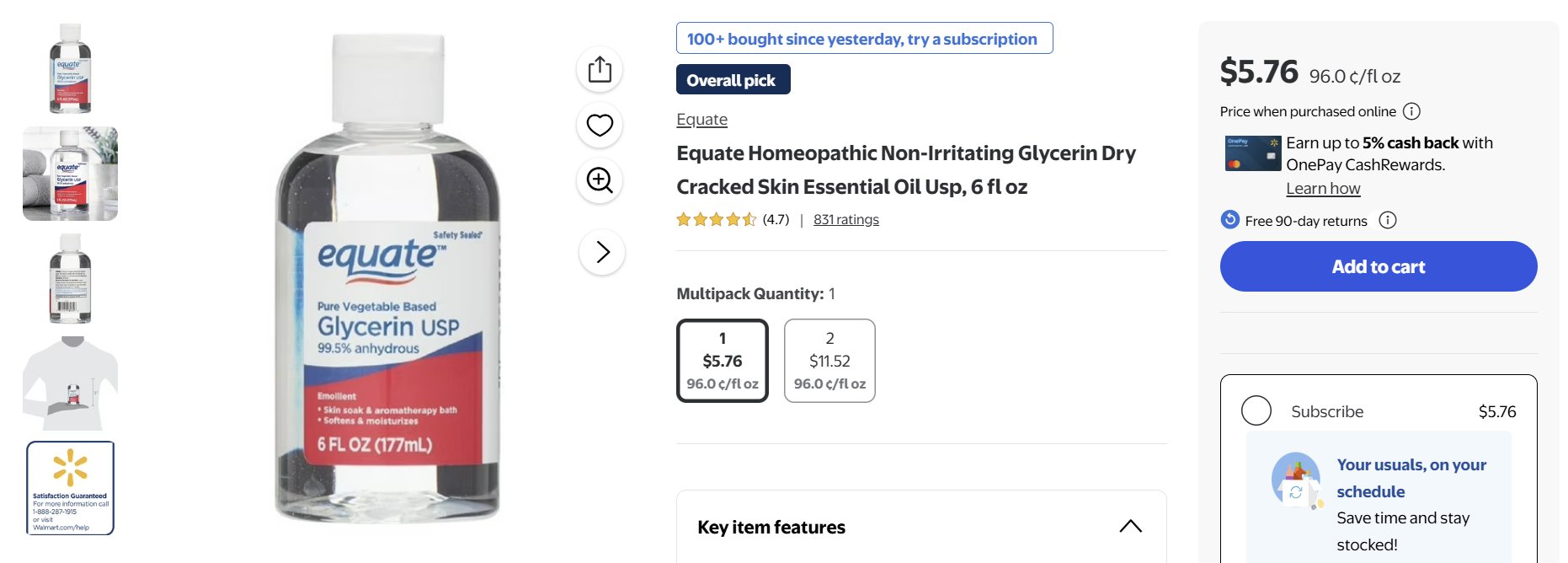 | 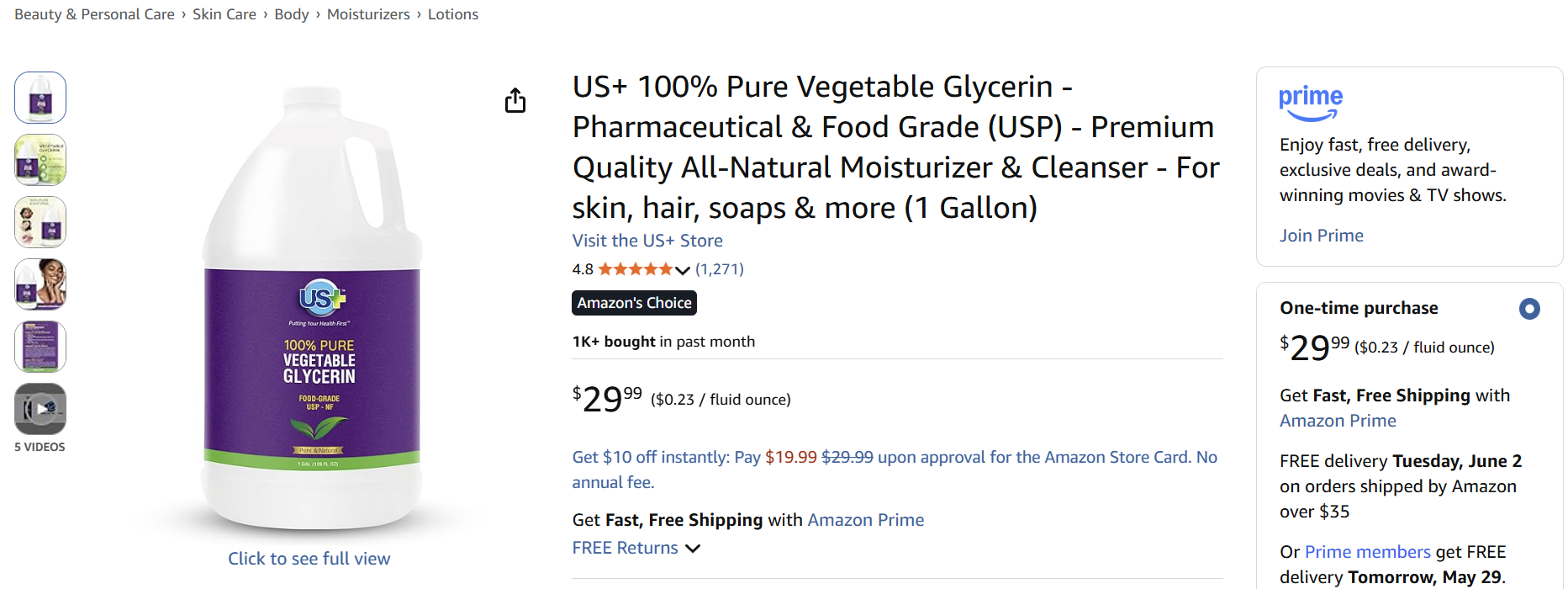 |
| --- | --- |
| **E. Glycerol, small retail: Walmart Equate Vegetable Glycerin, 6 fl oz, $5.76** | **F. Glycerol, Amazon/bulk: US+ Vegetable Glycerin, 1 gallon, $29.99** |

| 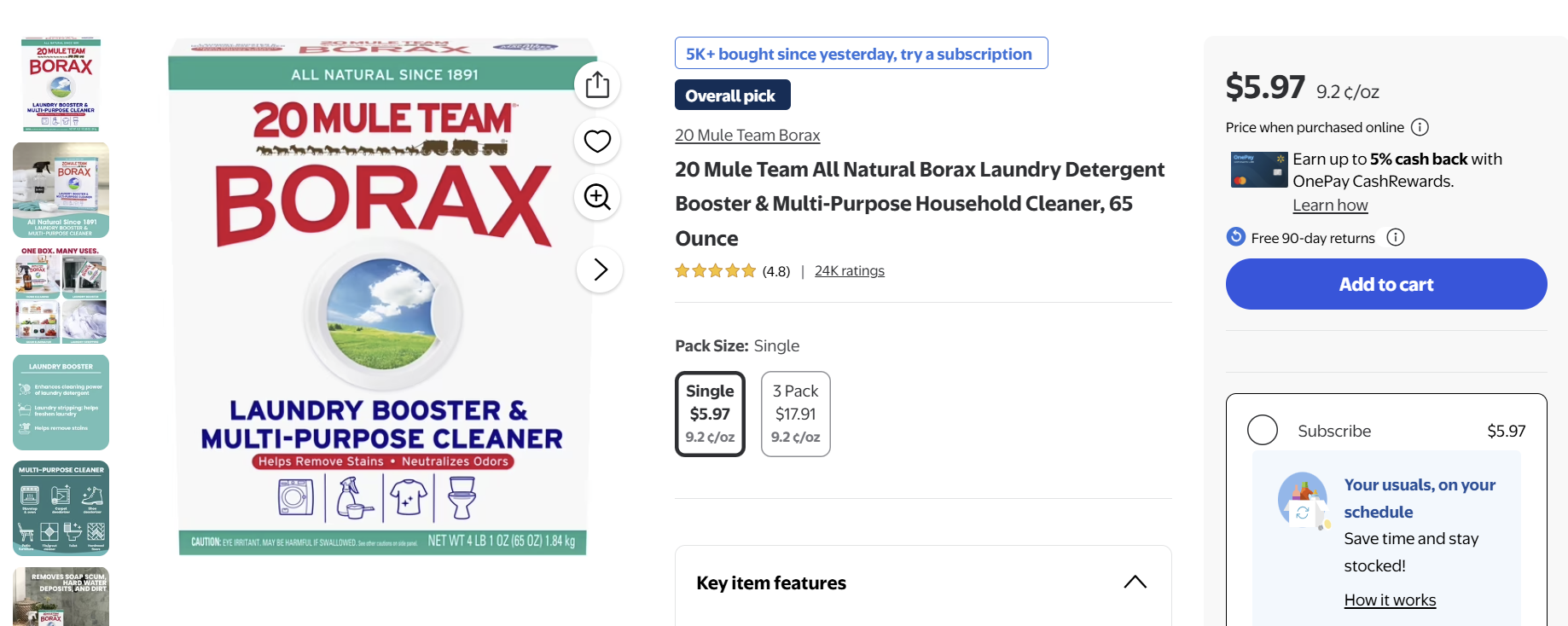 | 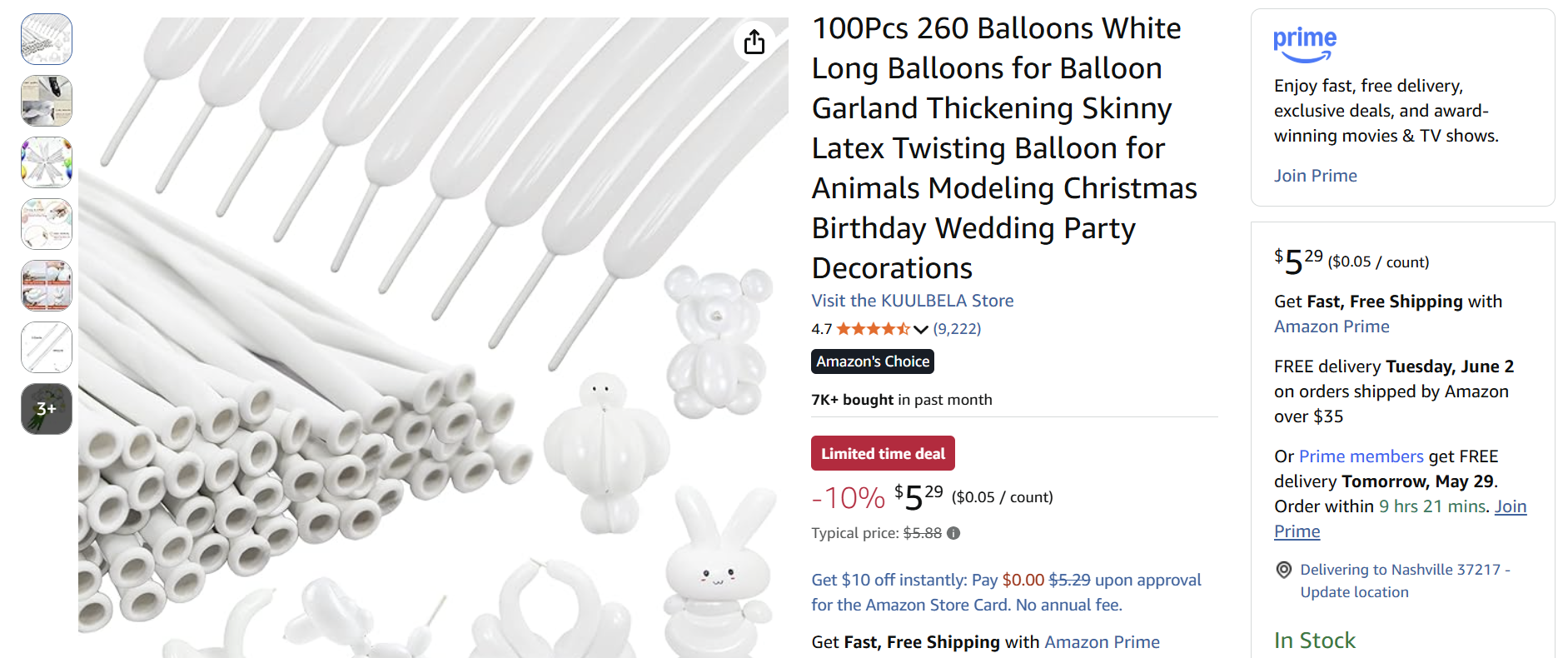 |
| --- | --- |
| **G. Borax powder: Walmart 20 Mule Team Borax, 76 oz, $5.97** | **H. 260 balloon: Amazon 100Pcs 260 Balloons, $5.29** |

| 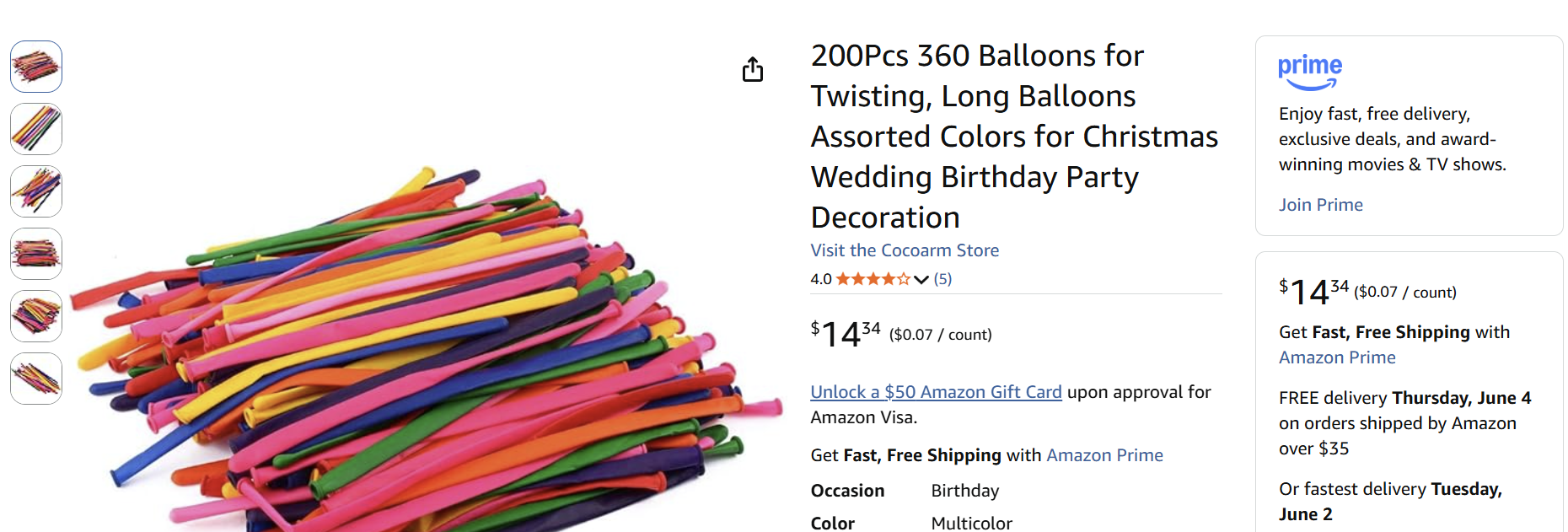 |  |
| --- | --- |
| **I. 360 balloon: Amazon 200Pcs 360 Balloons, $14.34** |  |
